# Vladislav Dzerzhinsky (1881–1942)

**DOI:** 10.1007/s10072-024-07679-2

**Published:** 2024-07-04

**Authors:** Sławomir Gonkowski, Maciej Kowalczyk, Krystyna Makowska

**Affiliations:** 1 Department of Clinical Physiology. Faculty of Veterinary Medicine, University of Warmia and Mazury, Olsztyn, Poland; 2 Gabinet Weterynaryjny Vet-Com, Olsztyn, Poland; 3 Department of Clinical Diagnostics, Faculty of Veterinary Medicine, University of Warmia and Mazury, Olsztyn, Poland

Vladislav Dzerzhinsky was born in Polish impoverished noble family, on March 1st [1] or 4th [2], 1881 on the estate Oziembłowo (later renamed Dzierżynowo) located not far to the west of Minsk – the capital of to-day Belarus [1, 2]. The interesting fact is that he was a younger brother of Felix Dzerzhinsky – nicknamed “Iron Felix” – later Bolshevik revolutionary and official, one of the architects of the red terror in Russia after October Revolution [3].

Vladislav Dzerzhinsky attended the secondary school in Vilnius, and then in Saint Petersburg [3]. In 1900 he started to study medicine at Moscow University, receiving his medical degree in 1905 [1] or 1906 [3]. Then he worked in the Clinics of Neurological Diseases at the Moscow University directed by Vladimir Roth—the most eminent Russian neuropathologist and neurologist of that period. In 1911 Vladislav Dzerzhinsky obtained a doctorate in medicine based on dissertation “Onto-, phylo- and histogenesis of adrenal medulla” [1, 3], and in 1913 he started to work in the local Provincial Hospital in Kharkov (now in Ukraine), which at that time was the leading and largest medical institution in the Russian Empire [3]. During the first period of the World War I Vladislav Dzerzhinsky served as military doctor in the Russian army [3], but soon he came back to returned Kharkov and in 1915 he was awarded the title of assistant professor at the local university [1, 2].

In 1919 Vladislav Dzerzhinsky moved to Yekaterinoslav (currently Dnipro in Ukraine) and took part in the organization of newly established university [3]. In the same year he obtained the degree of full professor of medicine and assumed the position of the head of the Department of Nervous and Mental Diseases. In 1920 Vladislav Dzerzhinsky was elected dean of Medical Faculty, and in 1921 he was a prorectors of Yekaterinoslav University.

However, in 1922 Vladislav Dzerzhinsky emigrated from Soviet Russia to Poland. The reasons of this decision are not clear, but probably Vladislav Dzerzhinsky was an opponent of the “red terror” introduced in Russia (among others by his brother Felix) and saw no chance for his further scientific development under such conditions [1–3]. In Poland Vladislav Dzerzhinsky joined the Polish army. He served as a military doctor in hospitals in Cracow, Przemyśl and Łodź. In Łódź Dzerzhinsky became the head of the Department of Neurological Diseases in the modern, newly built Hospital of Social Security. In 1930 Vladislav Dzerzhinsky was promoted to the rank of colonel [2], and in 1934 he retired from the Polish army. However, he still worked in the hospital and held a private neurological practice [1]. Some sources record that Dzerzhinsky treated the poorest patients for free [2].

Vladislav Dzerzhinsky tragically died during occupation of Poland by Nazi Germany during the World War II. In February 1942 he was arrested by Nazi German authorities and shot on March 20th, 1942 in public execution in Zgierz, not far from Łódź. He was buried in a common grave in the forest in the vicinity of Zgierz [1, 2].

Scientific interest of Vladislav Dzerzhinsky were very diverse, which are reflected in his numerous scientific works. Vladislav Dzerzhinsky is the author of over 100 scientific articles [2]. First of all he is known as an author of the first description (published in 1913) of familiar hyperplastic periosteal dystrophy (dystrophia periostalis hyperplastica familiaris) known also in honor of discoverer as "Dzerzhinsky's syndrome” [4]. In the study Dzerzhinsky described insightful and comprehensive multidirectional clinical and anthropological observations, as well as family medical history in changes within the skeleton conducted on large families (for example in one family he analyzed changes in above 20 people), in which describing disease occurred. Dzerzhinsky also paid attention to the lifestyle of the patients (smoking, drinking alcohol), number of pregnancies in women and the diseases, from which the patients suffered in the past. He documented his research using photography and a relatively new method at the time—X-rays (discovered in 1895) (Fig. 1). Based on his own observations and a thorough analysis of previous works describing similar disorders, Dzerzhinsky precisely described the typical symptoms of the dystrophia periostalis hyperplastica familiaris and underlined that it is a congenital and family disease. According to Dzerzhinsky, the main symptoms of the disease include: premature synostosis of the cranial sutures and a fontanelle, thickening and sclerosis of the skull bones, skull size reduction, lordosis of the skull base, massive bone structure with thickening of long and short bones, well visible excessive thickness of the collarbones, brachyphalangy (e.i a shortening of the phalanges of the fingers) and pectus excavatum. The next symptoms were changes in the appearance of the face and nose and skull deformation, which depended on the order in which the sutures ossified. Simultaneously, Dzerzhinsky did not find any growth disorders during the course of the disease. It should be underlined that Dzerzhinsky did not know the causes of the described disease, which he saw in changes in the general metabolism resulting from hormonal disorders [4].

**Fig. 1:**
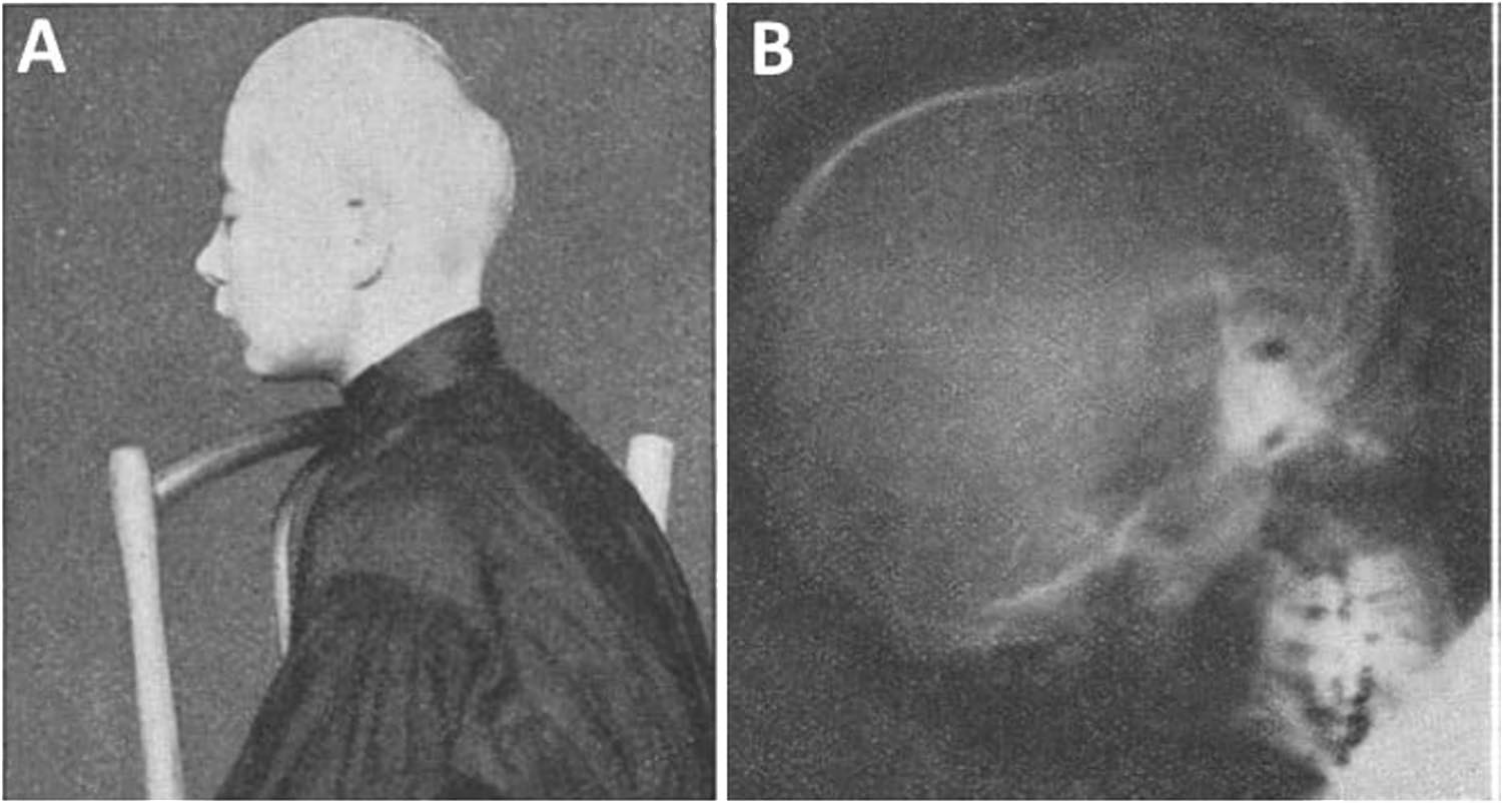
The patient suffering from Dzerzhinsky's syndrome (A) and roentgenogram of his skull (B) documented by Vladislav Dzerzhinsky and publicated in the work “Dystrophia periostalis hyperplastica familiaris” in 1913 (Z. f. d. g. Neur. u. Psych. 20: 547–560)

The next disease, which was the subject of Dzerzhinsky’s studies was myoclonus. Dzerzhinsky together with A. Kozhevnikov in article entitled “Ocoбaя фopмa ceмeйнoй миoклoнии” (eng. A special form of familial myoclonus) published in 1911 in Russian journal “Жypнaл нeвpoлoгии и пcиxиaтpии им. C. C. Кopcaкoвa” (T.XI, p.222) for the first time described one of the types of myoclonus under the Latin name „myoclonia familiaris nocturno-atactica” [5]. Interestingly, a precise identification of the identity of the co-author of this article is problematic, because famous neurologist Aleksei Kozhevnikov died in 1902. Therefore, it is not known whether the co-author of the above-mentioned article is another person with the same surname or whether it is a late publication of observations conducted before the death of Aleksei Kozhevnikov. The authors observed described disease in two generation of the same family, and therefore concluded that it was a congenital disease passed down from generation to generation. The diseases described by Dzerzhinsky was characterized by myoclonic twitches during sleep with mild ataxic phenomena in the lower limbs [5]. However, Dzerzhinsky did not know the etiology of the disease, nor did he propose methods of treating it. That's not surprising, because currently, it is known that this type of syndromes known as myoclonus-ataxia syndromes (MAS) may be caused by over 100 reasons [6]. MAS usually have genetic basis and determination of their etiology is impossible to identify without advanced genetic testing. Moreover, even today, recognize these syndromes, determination their etiology and effective treatment often pose many difficulties [6]. In older Russian-language literature, the type of myoclonus described by Dzerzhinsky is sometimes called “Dzerzhinsky's disease” or “Dzerzhinsky's syndrome” [3]. However, it is not an eponym used in specialized international literature.

In his multidisciplinary research work, Dzerzhinsky also dealt with a wide range of other neurological diseases. Among others, he wrote about symptoms of epilepsia partialis continua described for the first time by Russian neurologist Aleksei Kozhevnikov [7] and Unverricht–Lundborg disease – one of forms of progressive myoclonus epilepsies [8]. The next interesting Dzerzhinsky's work was the description of defense reflexes after damage to the spinal cord [9]. In this article, Dzerzhinsky used his experience as a military doctor during the World War I regarding the treatment of soldiers after gunshot wounds to the spine. Changes in defense reflexes, in which reflexes partially disappear but some of their elements remain unchanged, Dzerzhinsky described as “splitting of defense reflexes”.Vladislav Dzerzhinsky is also the author of the first Polish book concerning neurology—“A textbook of nervous diseases” (volume 1 published in 1925, volume 2 in 1927) in which both physiology of the nervous system and neurological diseases were presented in a comprehensive and modern way [10, 11].

Vladislav Dzerzhinsky deserves to be remembered not only as a talented scientist, pioneer and professor of neurology, but also as a practicing physician helping his patients.

## Conflicts of interest:

The authors state that there is no conflict of interest.
